# Optimization of bacteriophage therapy for difficult-to-treat musculoskeletal infections: a bench-to-bedside perspective

**DOI:** 10.3389/fcimb.2024.1434397

**Published:** 2024-09-03

**Authors:** Laura Bessems, Baixing Chen, Saartje Uyttebroek, David Devolder, Cédric Lood, Stefaan Verwimp, Paul De Munter, Yves Debaveye, Melissa Depypere, Isabel Spriet, Laura Van Gerven, Lieven Dupont, Jeroen Wagemans, Vera van Noort, Rob Lavigne, Willem-Jan Metsemakers, Jolien Onsea

**Affiliations:** ^1^ Department of Development and Regeneration, KU Leuven, Leuven, Belgium; ^2^ Department of Trauma Surgery, University Hospitals Leuven, Leuven, Belgium; ^3^ Department of Neurosciences, Experimental Otorhinolaryngology, Rhinology Research, KU Leuven, Leuven, Belgium; ^4^ Department of Otorhinolaryngology, University Hospitals Leuven, Leuven, Belgium; ^5^ Pharmacy Department, University Hospitals Leuven, Leuven, Belgium; ^6^ Laboratory of Gene Technology, Department of Biosystems, KU Leuven, Leuven, Belgium; ^7^ Center of Microbial and Plant Genetics, KU Leuven, Leuven, Belgium; ^8^ Laboratory for Clinical Infectious and Inflammatory Disorders, Department of Microbiology, Immunology and Transplantation, KU Leuven, Leuven, Belgium; ^9^ Department of Internal Medicine, University Hospitals Leuven, Leuven, Belgium; ^10^ Department of Intensive Care Medicine, University Hospitals Leuven, Leuven, Belgium; ^11^ Department of Laboratory Medicine, University Hospitals Leuven, Leuven, Belgium; ^12^ Laboratory of Clinical Microbiology, Department of Microbiology, Immunology and Transplantation, KU Leuven, Leuven, Belgium; ^13^ Department of Pharmaceutical and Pharmacological Sciences, Clinical Pharmacology and Pharmacotherapy, KU Leuven, Leuven, Belgium; ^14^ Department of Microbiology, Immunology and Transplantation, Allergy and Clinical Immunology Research Group, KU Leuven, Leuven, Belgium; ^15^ Department of Pneumology, University Hospitals Leuven, Leuven, Belgium; ^16^ Department of Chronic Diseases and Metabolism, Respiratory Diseases and Thoracic Surgery, KU Leuven, Leuven, Belgium; ^17^ Institute of Biology, Leiden University, Leiden, Netherlands

**Keywords:** bacteriophages, bacteriophage therapy, treatment optimization, bench-to-bedside, musculoskeletal infections

## Abstract

Given the increasing threat of antimicrobial resistance, scientists are urgently seeking adjunct antimicrobial strategies, such as phage therapy (PT). However, despite promising results for the treatment of musculoskeletal infections in our center, crucial knowledge gaps remain. Therefore, a prospective observational study (PHAGEFORCE) and a multidisciplinary approach was set up to achieve and optimize standardized treatment guidelines. At our center, PT is strictly controlled and monitored by a multidisciplinary taskforce. Each phage treatment follows the same pathway to ensure standardization and data quality. Within the PHAGEFORCE framework, we established a testing platform to gain insight in the safety and efficacy of PT, biodistribution, phage kinetics and the molecular interaction between phages and bacteria. The draining fluid is collected to determine the phage titer and bacterial load. In addition, all bacterial isolates are fully characterized by genome sequencing to monitor the emergence of phage resistance. We hereby present a standardized bench-to-bedside protocol to gain more insight in the kinetics and dynamics of PT for musculoskeletal infections.

## 1 Introduction

The history of treating musculoskeletal infections (MSIs) with bacteriophages stretches back over 100 years. Especially in the former Soviet Union, the research and development of phage therapy (PT) for this indication has continued over the last century. With the looming threat of antimicrobial resistance (AMR), the interest in PT has expanded to countries outside the former Soviet Union as well (Luong et al., 2020; Moelling et al., 2018). A recent systematic review summarizes clinical data of the past 20 years on the safety and efficacy of phage therapy for difficult-to-treat infections (Uyttebroek et al., 2022). Promising clinical outcomes have been reported for the treatment of MSIs with phages. However, it is important to note that the number of case series and reports clearly outweighed the number of randomized controlled trials in this field. Furthermore, all studies applied different treatment or administration protocols, using different phages, which makes it difficult to draw conclusions regarding the optimal treatment protocol. The lack of treatment guidelines or a standardized treatment protocol can be attributed to the high abundance and variety of different phages and a lack of knowledge on phage kinetics (e.g. phage biodistribution, systemic effects). In addition, the self-amplification of phages in the presence of host bacteria leads to fluctuating phage densities which are not easy to predict or monitor. This is different from antibiotic therapy, where precise dosing regimens are based on decades of basic research and clinical trials (Metsemakers et al., 2023).

In Belgium, a groundbreaking framework for the utilization of phages as magistral preparations was approved in 2018. Magistral preparations refer to ‘any medicinal product prepared in a pharmacy in accordance with a medical prescription for an individual patient’. In this framework, a monograph defines the characteristics and quality standards of phage active substances for human applications. Compliance with these standards is evaluated by a Belgian Approved Laboratory (i.e., the Belgian Institute for Health, Sciensano) (Pirnay et al., 2018). Following these developments and to promote standardization, the PHAGEFORCE study was set up in our center, which prospectively collects data on the treatment and outcomes of patients with difficult-to-treat musculoskeletal infections (MSI), chronic rhinosinusitis, sepsis, pulmonary infections and hidradenitis suppurativa (Onsea et al., 2021b). In this framework, a multidisciplinary team, referred to as the Coordination group for Bacteriophage therapy Leuven (CBL), was established. The aim of this manuscript is to summarize the CBL’s perspective and experience regarding PT for difficult-to-treat MSI.

## 2 A bench-to-bedside approach to the application of phage therapy

**Figure 1:**
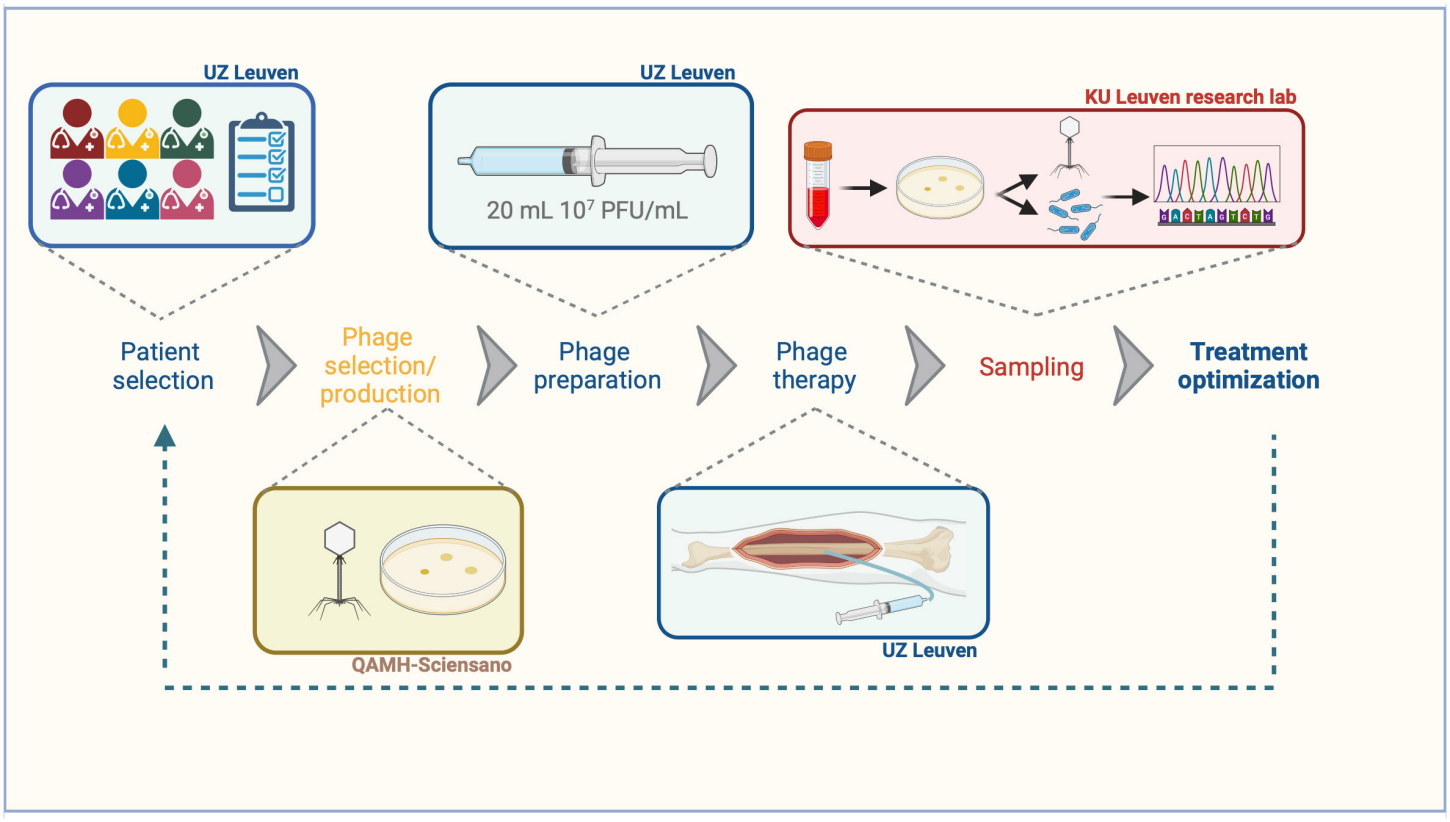
Bench-to-bedside approach aiming to optimize phage therapy. Blue: actions that are performed at the University Hospitals Leuven. Yellow: actions that are performed at the Queen Astrid Military Hospital in collaboration Sciensano. Red: actions that are performed in a research lab.

Our PT approach is a standardized, multi-step process which requires an interdisciplinary collaboration between different hospitals and research labs (**Figure 1**). The ultimate goal is to create a continuous feedback loop to further optimize our treatment protocols.

### 2.1 Patient eligibility

**Figure 2:**
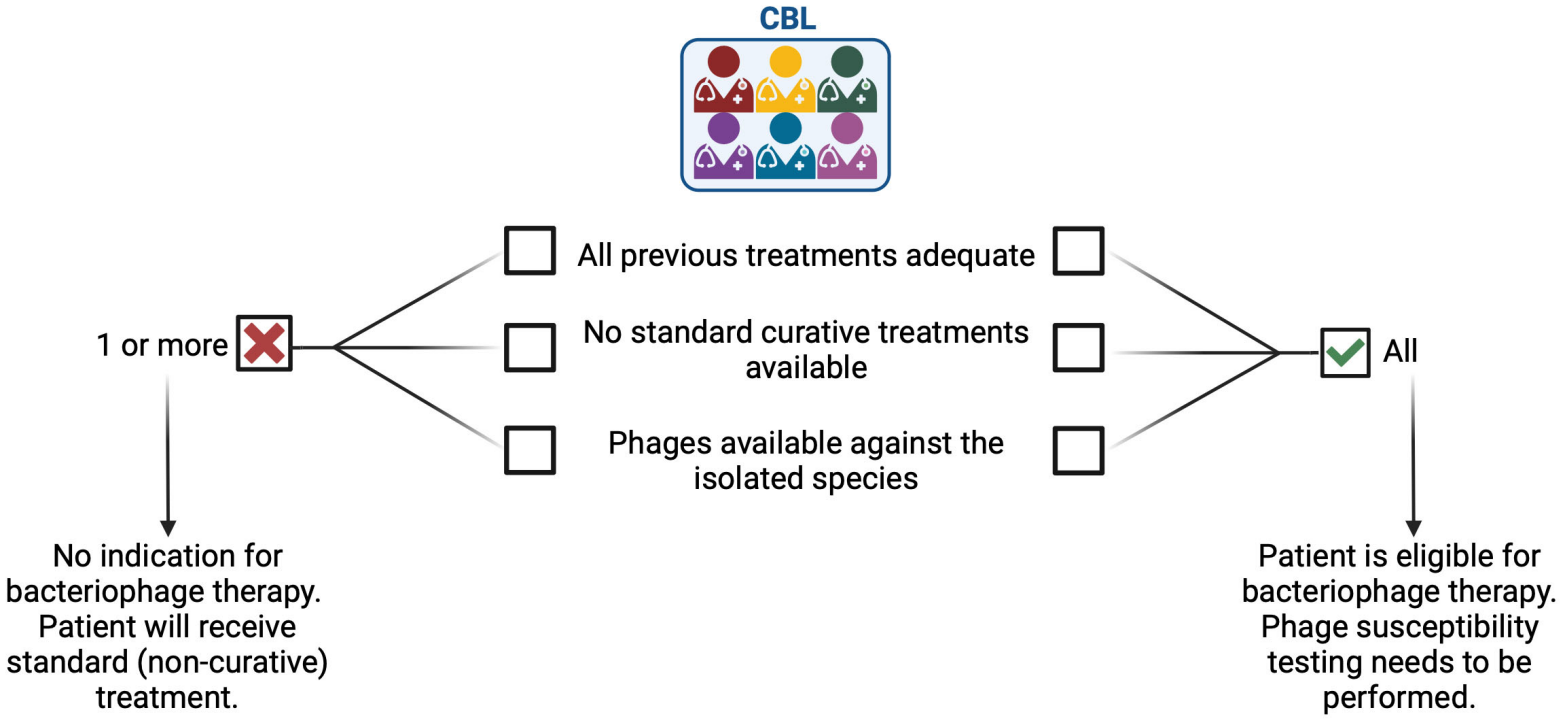
Patient eligibility for phage therapy is determined by a multidisciplinary team.

The CBL consists of trauma surgeons, otorhinolaryngologists, pneumologists, dermatologists, intensive care physicians, infectious disease specialists, microbiologists, hospital pharmacists, phage researchers and coordinators. Each application for PT is thoroughly reviewed by the CBL to evaluate eligibility. Patients are eligible for PT when all previous treatments were adequate and no other standard (curative) treatments are available (**Figure 2**). If eligible and if phages are available against the causative pathogens, the patient will be asked to sign informed consent to have his/her data collected in a patient registry. Initially, if not already available, intraoperative deep cultures are obtained from the infection site, allowing for the identification of the causative pathogen(s). The susceptibility of the pathogen(s) against the available phages is then determined by means of a phagogram (see below). If active phages are available, the CBL provides the treatment and sampling protocol to ensure the collection of standardized data. Each patient is followed and monitored by a dedicated PT coordinator during and after treatment.

### 2.2 Phage selection

If the CBL deems the patient eligible for PT, a phagogram is determined on the isolated pathogens. Similar to an antibiogram, a phagogram determines the susceptibility of the isolated pathogen(s) against an available range of phages. Reference methods used to perform the phagogram are the double agar overlay plaque assay and the spot test (Daubie et al., 2022). A measure for the relative activity of a phage against the isolated pathogen as compared to its reference host strain is the efficiency of plating (EOP). The higher the EOP, the more susceptible the tested strain is relative to the phage’s reference host strain. When the EOP is below 0.001, the phage is considered not suitable for treatment. In case multiple phages are available, the patient will receive a phage cocktail. In case of confirmed polymicrobial infections, only patients for whom all pathogens are susceptible and for which phages are available can be treated with PT. In case of a negative phagogram, the patients will receive standard (non-curative) treatment.

### 2.3 Phage production

In Belgium, phages are produced at the Queen Astrid Military Hospital (QAMH) according to the monograph defining the characteristics and quality standards of phage active substances for applications in humans (Pirnay et al., 2018). After production, a Belgian Approved Laboratory (Sciensano) evaluates whether the active substances are conform to the provisions of the monograph. Furthermore, for each phage (in combination with their production host), a genetic passport first needs to be approved, demonstrating the strictly lytic profile as well as the absence of toxins and antibiotic resistance genes. The phage Active Pharmaceutical Ingredient (API) consists of concentrated phage with a titer between 10^9^ and 10^10^ plaque forming units (PFU)/mL in Dulbecco’s phosphate buffered saline (DPBS) (pH ranging between 6.0 and 8.0). After batch approval and certification, the phage API is sent to the hospital pharmacy for further preparation.

### 2.4 Phage preparation

In a biological safety cabinet (BSC Class II), the concentrated phage API is diluted in 0.9% NaCl to a final concentration of 10^7^ PFU/mL and pH 5.5 (**Figure 1**). 20 mL solutions at 10^7^ PFU/mL are prepared in 20 mL polypropylene syringes (BD Plastipak, ref. 300629) and stored at 4°C before administration (**Figure 1**). Based on a recent stability study, these preparations expire after seven days, after which new dilutions are prepared to complete the 10-day treatment regimen (Uyttebroek et al., 2023). The hospital pharmacy compounds polypropylene syringes with 20 mL 1.4% sodium bicarbonate to boost the activity of phages in alkalic conditions (pH 7.0-8.5) before the administration of phage solution.

### 2.5 Treatment protocol

Patients with an MSI, for whom phages (with a genetic passport) are available, are routinely treated with a surgical debridement during which cultures are taken. After debridement, the surgical wound is rinsed with phage solution (in 0.9% NaCl). Subsequently, a draining system is placed at the infected site. After closing of the wound, the draining system is tested. First, 1.4% sodium bicarbonate is injected to create an alkaline environment, after which the phage solution is injected (titer 10^7^ PFU/mL). The injected volume depends on the location (affected limb) and defect size, and ranges between 20 to 40 mL, as determined by the surgeon. After injection of the phage solution, the draining tubes are clamped for 10 minutes after which the fluids are collected in the Redon collection system. Using the same administration protocol, at the hospital ward, the patient is treated through the draining system three times per day, for a total duration of 10 days. After each administration, a Redon bottle is connected to the drain to collect all fluid. Postoperative treatment is performed by the nursing staff. During treatment, the patient is monitored closely. Vital parameters are taken before and after each administration and blood analyses are performed regularly to monitor systemic effects, as detailed below (Sampling).

### 2.6 Sampling

The During treatment, draining fluid is collected daily, after each administration of phage solution, and placed at 4°C until analysis. In addition, blood samples are routinely collected prior to treatment, at days 1, 2, 4, 7, 10 and 14. Thereafter, blood samples are collected every two weeks until three months after phage treatment. Blood tests include determining the complete blood count, basic metabolic panel, inflammatory parameters, lactic acid, creatine kinase and liver function tests. Serum samples are stored at -80°C for a phage neutralization assay according to a modified Adams protocol, as previously described (Onsea et al., 2019).

In the research laboratory, serial dilutions of the draining fluid containing phages are prepared in phage buffer (10 mM Tris, 10 mM MgSO4, 150 mM NaCl, pH 7.5). Then, 100 µL of the diluted phage solutions are mixed with 200 µL of overnight grown host bacteria and 4 mL of soft LB (Lysogeny Broth, Becton Dickinson, Franklin Lakes, NJ, USA) medium with 0.6% agar (Becton Dickinson) at 45°C. The mixtures are then poured onto petri plates containing a solidified bottom layer of Trypticase soy agar (TSA) (Becton Dickinson) medium containing 1.5% agar. After air-drying, the plates are incubated overnight at 37°C. Plaques are observed the next day and the titer (PFU/mL) is determined. The bacterial load is determined by inoculating 200µL of sample on 5% horse blood agar plates (BA, Oxoid Ltd, Hampshire, United Kingdom). Plates are incubated at 37°C and colony forming units (CFUs) are quantified after 24 hours. Furthermore, to gain insight in resistance development of the bacterial strain against the applied phage, the EOP is determined for all bacteria isolated during and after (if applicable) phage treatment and compared to the initial (pre-treatment) one. In addition, all bacterial isolates collected before, during and after treatment (if available) are characterized by whole genome sequencing using Illumina and/or Nanopore sequencing (Lood et al., 2021). The genome content of infecting bacteria will be used to develop digital phagograms, that in the future may help identify effective phages that are not physically available at a specific location. The collected phages are sequenced using Illumina sequencing only. By comparing the resulting sequencing reads to the genome of the original isolate in a variant analysis, mutations in the phage genome that may increase phage efficiency in the patient environment or mutations in the bacterial genome that may result in a reduced EOP are identified. These insights will deepen our understanding of the molecular biology behind phage-bacteria interactions and guide the development of future phage cocktails for PT.

Finally, the patient’s pseudonymized data regarding treatment of the MSI including the patient’s clinical status during and after treatment is collected and stored using REDCap (Research Electronic Data Capture). REDCap is a secure, web-based software platform designed to support data capture for research studies, providing an interface for validated data capture, audit trails for tracking data manipulation and export procedures, automated export procedures for seamless data downloads to common statistical packages and procedures for data integration and interoperability with external sources (Harris et al., 2019, 2009).

## 3 Discussion

### 3.1 Local sampling to monitor phage – host interactions

The presented bedside administration protocol through a draining system is straightforward and provides the opportunity for sampling and real-time monitoring of phage and bacterial load. During the first application, which is performed intraoperatively, extra deep tissue cultures are taken. If these cultures reveal additional pathogens that were not cultured before (i.e. in case of chronic polymicrobial infections), adaptations in the phage cocktail or concomitant antibiotic therapy regimen may be required (Onsea et al., 2021b). After surgical placement of the draining system, phage therapy is applied at the patient’s bedside, in the hospital ward. An advantage of such a direct application approach is that the phage titer can be determined as well as the bacterial load during treatment. Phage titer determination in the draining fluid is primarily important to observe the interactions between the administered phages and the host bacteria. Furthermore, by determining the phage titer on a daily basis, we obtain a clear understanding of the stability of the phages throughout the treatment and are able to act accordingly. If the titer drops significantly, this could potentially be attributed to sampling, but if this drop is consistent, other causes like problems with the draining system should be investigated. To monitor treatment progress, the bacterial load in the draining fluid is determined daily. In case the bacterial load systematically increases during treatment, this could indicate phage resistance, which is then investigated further in the research lab.

A disadvantage of the instillation of phages through a draining system is that it requires multiple administrations and the patient needs to remain hospitalized for the duration of treatment. Another disadvantage is the potential risk of superinfection through the draining system. To reduce this risk, PT is only applied by experienced nurses and the drain is removed as soon as possible after treatment.

### 3.2 Optimization

By collecting and whole genome sequencing of all bacterial and phage isolates, we aim to detect ‘in patient-optimized’ phages. Some mutations in the phage structural proteins might result in an increased stability within the patient. These phages will then form the foundation for next-generation therapeutic phages that could be used in personalized PT. Additionally, mutations in the bacterial genome resulting in a decreased EOP are deciphered. This will guide us in the development of future phage cocktails (what phage types should be combined to reduce resistance development) or combination therapies with antibiotics. Indeed, phage resistance is often accompanied with antibiotic sensitivity due to fitness trade-offs (Fujiki et al., 2023). Ultimately, to recognize the critical importance of bridging the gap between bench and bedside, a feedback loop (**Figure 1**) is established that connects all genomic insights with clinical practice. This approach ensures that the data derived from sequencing efforts are not solely academic but also inform clinical decision-making, ensuring tailored treatment regimens that maximize efficacy and safety for each patient.

### 3.3 Future perspective

A significant limitation of phage therapy is the host range of phages as well as the fact that bacteria can develop resistance to phages. Phage training is the process of evolving phages to target specific bacteria or improve their effectiveness against resistant strains. Recent reports show promising outcomes using this approach (Friman et al., 2016; Eskenazi et al., 2022). However, the concept of phage training for treatment purposes is still in early stages with limited clinical data. Further research is needed to develop protocols, understand trained phages *in vivo*, and assess long-term safety and benefits. As research progresses, phage training could become a key part of personalized medicine for challenging bacterial infections (Rohde et al., 2018).

Combining phage therapy with adequate antibiotic therapy is a critical aspect of our treatment strategy. This approach takes advantage of the synergistic effects of both treatments, aiming to enhance overall efficacy (Ferry et al., 2024). The choice of antibiotic is guided by the antibiogram and standard antibiotic use guidelines. Evidence suggests that phage therapy is more effective when used alongside systemic and/or local antibiotic therapy (Wang et al., 2020). Additionally, combination therapy is associated with a reduced risk of resistance development. Bacterial resistance to phages typically involves modifications to cell surface receptors, which impede phage adsorption but can increase susceptibility to antibiotics (Levin and Bull, 2004; Maciejewska et al., 2018; Rohde et al., 2018). Conversely, antibiotics inhibiting bacterial protein synthesis can disrupt the phage life cycle, reducing phage replication (Zuo et al., 2021). Synergistic or antagonistic interactions between phages and antibiotics are highly dependent on the bacterial strain, the phage and the mechanism of bacterial inhibition of the antibiotic (Onsea et al., 2021a). However, at this moment, there is insufficient knowledge to predict phage-antibiotic interactions prior to treatment. Further research is required to develop and validate a testing platform to determine phage-antibiotic synergy.

## 4 Conclusion

PT is a promising treatment option for difficult-to-treat MSI, but because of the crucial knowledge gaps a multidisciplinary approach is important. Furthermore, we highlight the importance of local sampling to monitor phage – host interaction.

We hereby present a standardized and sustainable bench-to-bedside approach to monitor local phage titers and bacterial load to gain more insight in PT kinetics and dynamics.

## Data Availability

The original contributions presented in the study are included in the article/supplementary material. Further inquiries can be directed to the corresponding author.
